# Transcriptome Analysis Reveals Increases in Visceral Lipogenesis and Storage and Activation of the Antigen Processing and Presentation Pathway during the Mouth-Opening Stage in Zebrafish Larvae

**DOI:** 10.3390/ijms18081634

**Published:** 2017-07-31

**Authors:** Hao Xu, Enxiu Liu, Yun Li, Xiaojie Li, Chenyu Ding

**Affiliations:** 1 College of Animal Science and Technology, Southwest University, Chongqing 400715, China; Xuhao@email.swu.edu.cn (H.X.); liuenxiu93@email.swu.edu.cn (E.L.); lxj0125@email.swu.edu.cn (X.L.); dcy866qq@emial.swu.edu.cn (C.D.); 2 The Key Laboratory of Freshwater Fish Reproduction and Development (Ministry of Education), Southwest University, Chongqing 400715, China; 3 Institute of Three Gorges Ecological Fisheries of Chongqing, Chongqing 400715, China

**Keywords:** mouth-opening stage, energy storage, zebrafish larvae, transcriptomic analysis, differentially expressed genes

## Abstract

The larval phase of the fish life cycle has the highest mortality, particularly during the transition from endogenous to exogenous feeding. However, the transcriptional events underlying these processes have not been fully characterized. To understand the molecular mechanisms underlying mouth-opening acclimation, RNA-seq was used to investigate the transcriptional profiles of the endogenous feeding, mixed feeding and exogenous feeding stages of zebrafish larvae. Differential expression analysis showed 2172 up-regulated and 2313 down-regulated genes during this stage. Genes associated with the assimilation of exogenous nutrients such as the arachidonic acid metabolism, linoleic acid metabolism, fat digestion and absorption, and lipogenesis were activated significantly, whereas dissimilation including the cell cycle, homologous recombination, and fatty acid metabolism were inhibited, indicating a physiological switch for energy storage occurred during the mouth-opening stage. Moreover, the immune recognition involved in the antigen processing and presentation pathway was activated and nutritional supply seemed to be required in this event confirmed by qPCR. These results suggested the energy utilization during the mouth-opening stage is more tended to be reserved or used for some important demands, such as activity regulation, immune defense, and lipid deposition, instead of rapid growth. The findings of this study are important for understanding the physiological switches during the mouth-opening stage.

## 1. Introduction

In oviparous fish, the mouth-opening stage is a crucial physiological switch period [1]. During this phase, fish larvae undergo a rapid ontogenetic switch in nutritional strategies from endogenous feeding (utilization of only the yolk-sac reserves) via mixed feeding (simultaneous utilization of yolk reserves and exogenous food) to exogenous feeding (exclusively relying on external feeds) [2]. The change in the nutritional source is accompanied by significant morphological changes, including changes to the eyes [3], gills [4], fin rays [5], muscle tissue [6], and adipose tissue [7]. These changes facilitate the development of physiological capacities that enable the fish to manage various environmental stresses at later stages, such as salinity, temperature, prey and predators [8]. Moreover, with the development of the digestive tract, rapid ontogenesis occurs, which promotes microflora colonization in the gut [9]. Studies have shown that host and microflora are interdependent. The majority of species cannot be survival when removed from their niches [10]. Conversely, the host takes advantage of the special functions of the microflora to complete certain essential physiological processes in the early mouth-opening stage, including angiogenesis [11], epithelial renewal [12], immune system development [13], and nutrient absorption [14]. Overall, oviparous fish have developed methods of supporting many physiological functions during the transition from the endogenous to the exogenous feeding phase. Nevertheless, compared with other developmental phases, fish larvae at the mouth-opening stage are more vulnerable, and associated with high mortality [1,15]. In aquaculture practice, hunger stress as one of the most common lethal factors during the mouth-opening stage has been avoided as far as possible [16]. We believe that the reason for the high mortality at the mouth-opening stage is closely related to the physiological characteristics in this period. These characteristics are involved in the establishment of the immune system, the maturation of assimilation function and the accumulation of macromolecular energy resources. However, the details of the molecular regulation process associated with energy metabolism, immune response and growth regulation are poorly understood in this phase.

The development of high-throughput techniques, such as RNA-seq, prompted us to explore the molecular mechanisms associated with the transition that occurs during the mouth-opening stage in oviparous fish. The understanding of physiological changes in this process is not only a problem involved in developmental biology, but also is directly related to aquaculture. Because of the rapid development, short generation time and easy genetic manipulation of zebrafish, this species is applied widely as a model organism in many different research fields [17,18,19,20,21]. Abundant genetic resources and biological data have been accumulated for this species; thus, zebrafish exhibit many advantages over other fish, especially the availability of comprehensive data on this species’ molecular mechanisms. Similar to other oviparous fish, zebrafish undergo a rapid switch in nutritional strategies during their mouth-opening stage; thus, the study of the molecular mechanisms involved in this stage in zebrafish larvae will benefit the development of aquaculture technology.

In the present study, zebrafish was used as a model to investigate the molecular regulation of the transition from endogenous to exogenous feeding. RNA-seq was used to select three sample populations: the endogenous feeding group (ENDO) at 96 h post fertilization (hpf), mixed feeding group (MIX) at 120 hpf, and exogenous feeding group (EXO) at 192 hpf. The sampling points were determined by the clear differentiation of nutritional sources. At 96 hpf, the fish larvae start swimming after hatching and yolk is the exclusive nutritional source of development. At 120 hpf, yolk-sac reserves and exogenous food sources are consumed concurrently. At 192 hpf, the yolk-sac reserves have been completely consumed and exogenous foods are the exclusive source of nutrition. By comparing the gene expression profiles of the ENDO, MIX, and EXO groups and performing further quantitative real time polymerase chain reaction (PCR) validation, the energy utilization, growth regulation and immune responses were found to change most strikingly during the mouth-opening stage. An intrinsic physiological switch during the mouth-opening stage was observed in zebrafish larvae that caused the build-up of lipid reserves via the increased uptake and reduced metabolism of fatty acids. Moreover, the antigen recognized pathway was also significantly activated following mouth opening, and the qPCR analysis confirmed that the nutritional supply was required. These data provide a framework for future investigations of the mechanisms underlying the immune regulation and energy distribution processes that occur during the transition from the endogenous to exogenous feeding stages, and such insights will be beneficial for the fish farming industry.

## 2. Results

### 2.1. Mapping of RNA-Seq Reads to the Zebrafish Genome

**Figure 1:**
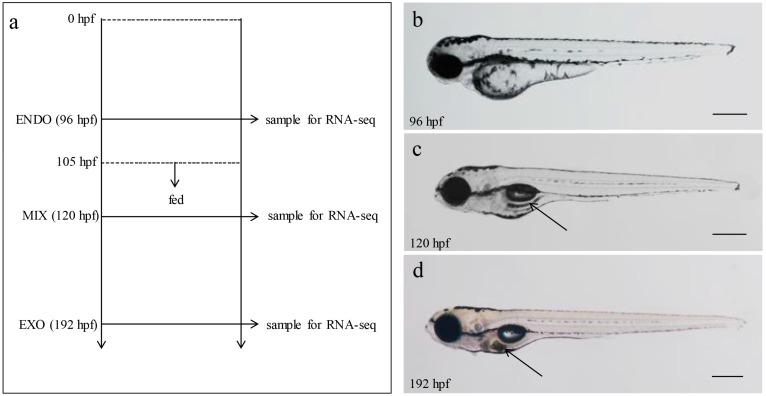
Samples were taken during transition from endogenous to exogenous feeding in zebrafish. (**a**) Flowchart of sampling. Zebrafish embryos were incubated at 28.5 °C from fertilization to 96 hpf, 120 hpf, and 192 hpf, respectively, and samples were taken for RNA-seq or qPCR. The time scales are shown on the left; (**b**) ENDO (96 hpf), the larvae start feeble swimming after hatching, yolk as the exclusive utilization of development; (**c**) MIX (120 hpf), the concurrent utilization of yolk reserves and exogenous feeds and (**d**) EXO (192 hpf), which is characterized by use of exclusively exogenous nutrition after the yolk was consumed completion. The black arrow annotated exogenous foods in intestinal tract. Bars = 400 μm in (**b**–**d**).

To reveal the transcriptional profiles for the transition from the endogenous to the exogenous feeding phase in zebrafish larvae, total RNA was extracted from two biological replicates each for the ENDO (96 hpf), MIX (120 hpf) and EXO (192 hpf) samples, and an RNA-seq analysis was performed (Figure 1a). Zebrafish larvae in the three phases displayed a distinguishable feeding behavior (Figure 1b–d). At 96 hpf, although the mouth had developed to a protrusion, feeding behavior was not observed. At 120 hpf, exogenous foods were observed easily in the intestinal tract, and the statistical feeding rate reached 100%. At 192 hpf, the yolk-sac reserves were exhausted, and the nutritional resources were exclusively exogenous.

RNA-seq generated 21.19–26.06 million (M) pairs of raw reads for each sample, and less than 3% of raw reads were discarded (Q < 20 and length < 25 bp) (Table 1). After the quality filtering, clean reads were extracted and mapped to the ribosome database. A total of 20.37 M–24.91 M reads were detected as the remaining reads and mapped to the zebrafish genome using *TopHat*. Moreover, 16.97 M–20.80 M reads were mapped to the total number of processed reads, and the ratio of unique mapping was 97.86–98.30% (Table 1).

### 2.2. Gene Expression Detected by RNA-Seq

**Figure 2:**
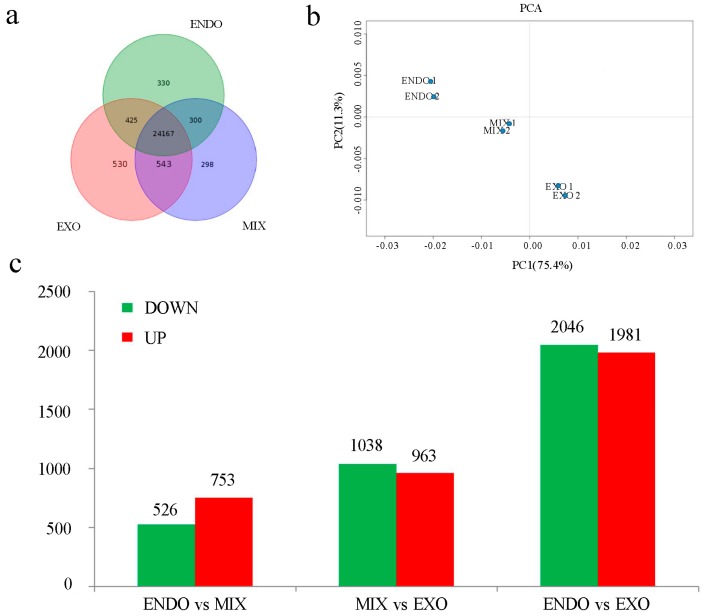
Bioinformatic analysis of RNA-seq data: (**a**) Venn diagram showing the developmental distribution of the genes detected; (**b**) principle component analysis (PCA) of gene expression in ENDO, MIX and EXO groups and (**c**) the number of differential expression genes between ENDO and MIX, ENDO and EXO, and MIX and EXO group.

In the present study, genes with a mean abundance >0 FPKM (Fragments per kilobase of transcript per million mapped reads) in any one of these samples were regarded as being expressed. According to Genome Reference Consortium Zebrafish Build 10 (GRCz10), 26,593 genes were detected during the mouth-opening stage (Figure 2a), and remaining 5422 genes were undetected. These non-detected gene transcripts were enriched in the gene ontology (GO) category biological processes (Table S1), which included neurological system processes, sensory perception of chemical stimuli, and G-protein coupled receptor signaling pathway. Moreover, 24,167 shared transcripts were observed among the three developmental stages. The number of transcripts shared only between two developmental stages was lower, with the ENDO stage sharing 300 transcripts with the MIX stage and 425 transcripts with the EXO stage (Figure 2a).

The overall change in gene expression during the transition from endogenous to exogenous feeding was analyzed by a principal component analysis (PCA). The inter- and intra-group variations in gene expression are shown in Figure 2b. The results showed that 86.7% of the variations in gene expression could be explained by the first two principal components (PCs). Minute variations in the intra-group were indicative of biological replicates highly. PCA projections of the ENDO, MIX and EXO groups in the PC space showed a significant distance, indicating that significant variations in gene expression occurred during the mouth-opening stage of larval development.

A pairwise comparison was performed among the ENDO, MIX, and EXO groups. The criteria of a two-fold or greater change in expression, *p*-value < 0.05 and false discovery rate (FDR) < 0.05, were used to determine the significantly up- or down-regulated genes. As a result, 1279 genes (753 up-regulated and 526 down-regulated) displayed a change in expression between ENDO and MIX, 2001 genes (963 up-regulated and 1038 down-regulated) between MIX and EXO, 4027 genes (1981 up-regulated and 2046 down-regulated) between ENDO and EXO (Figure 2c; Table S2). Moreover, we performed multiple comparisons using ENDO as the control and MIX and EXO as the treatments. A total of 4965 genes were identified as differentially expressed genes (DEGs) and used for further analysis (Table S3).

### 2.3. Analysis of Differential Expression

**Figure 3:**
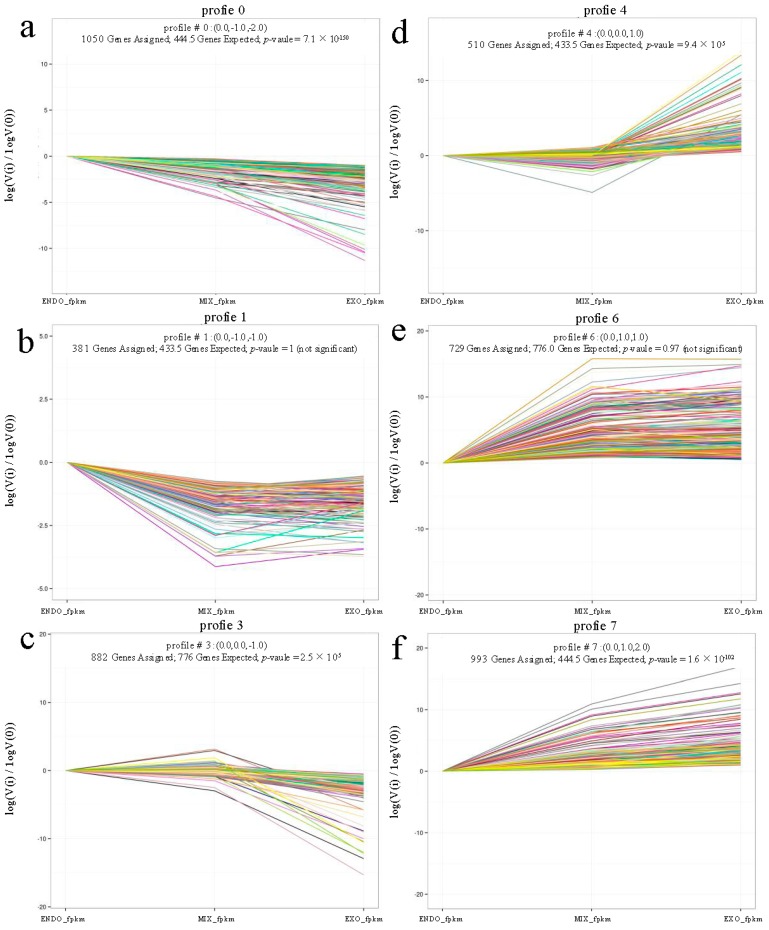
DEGs expression profiles from ENDO, MIX, and EXO: Profile 0 (**a**); Profile 1 (**b**); and Profile 3 (**c**) indicating down-regulated patterns; and Profile 4 (**d**); Profile 6 (**e**); and Profile 7 (**f**) indicating up-regulated patterns.

In the present study, to explore the physiological switches during the mouth-opening stage, we focused on the expression profiles of the 4965 DEGs. From ENDO to EXO, the expression data υ were normalized to 0, log_2_ (υMIX/υENDO), and log_2_ (υEXO/υENDO). The DEGs could be clustered into eight profiles using the short time-series expression miner (STEM). In total, 4485 DEGs could be clustered into the six profiles (*p*-value ≤ 0.05), including three down-regulated patterns in Profiles 0, 1, and 3 (Figure 3a–c), and three up-regulated patterns in Profiles 4, 6 and 7 (Figure 3d–f). Profiles 0, 1 and 3 contained 1050, 381 and 882 DEGs, respectively, while Profiles 4, 6 and 7 contained 510, 729 and 933 DEGs, respectively, (Figure S1).

**Figure 4:**
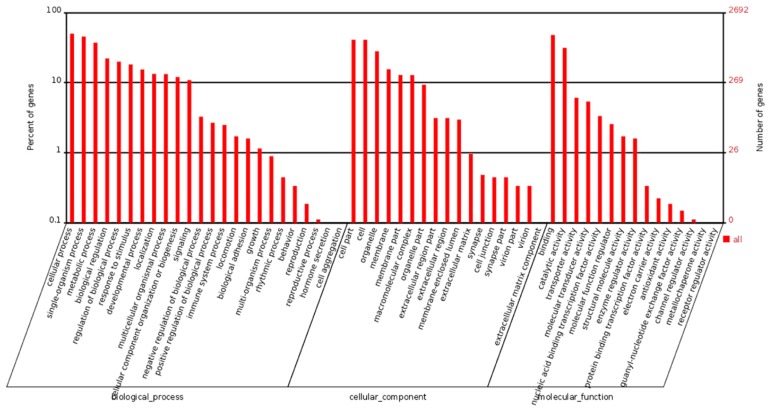
GO classification of DEGs. Biological process, cellular component and molecular function were analyzed.

Next, functional classification of the DEGs was performed to analyze the GO terms using Web gene ontology annotation plot (WEGO). Among the 4965 DEGs, WEGO provided functional annotation for 2692 genes, which were classified into three main functional clusters (biological process, cellular component, and molecular function), and more than 50 GO terms were overrepresented. The DEGs in the biological processes cluster were mainly related to stimuli responses, immune responses and developmental processes (Figure 4).

To identify the switch of physiological pathways during the mouth-opening stage, we mapped 4965 DEGs to the kyoto encyclopedia of genes and genomes (KEGG) database, and 36 pathways were overrepresented (Table S4). The top 10 KEGG pathways with the highest representation of DEGs are shown in Table 2. In the fat digestion and absorption pathways, 16 genes were found to be differentially expressed. The two genes annotated to Profile 4, six genes annotated to Profile 6 and eight genes annotated to Profile 7 showed different patterns of up-regulation, and none of the genes showed patterns of down-regulation. Similar results were observed in the antigen processing and presentation, arachidonic acid metabolism, and linoleic acid metabolism pathway. Conversely, the homologous recombination and cell cycle pathway, and fatty acid metabolism showed significant down-regulation trends (Table 2 and Table S4).

Overall, the results from the GO and KEGG pathway analyses showed that the DEGs were highly associated with immune response, growth regulation and energy utilization in zebrafish larvae during the mouth-opening stage.

### 2.4. Fat Digestion, Absorption and Fatty Acid Metabolism during the Mouth-Opening Stage

**Figure 5:**
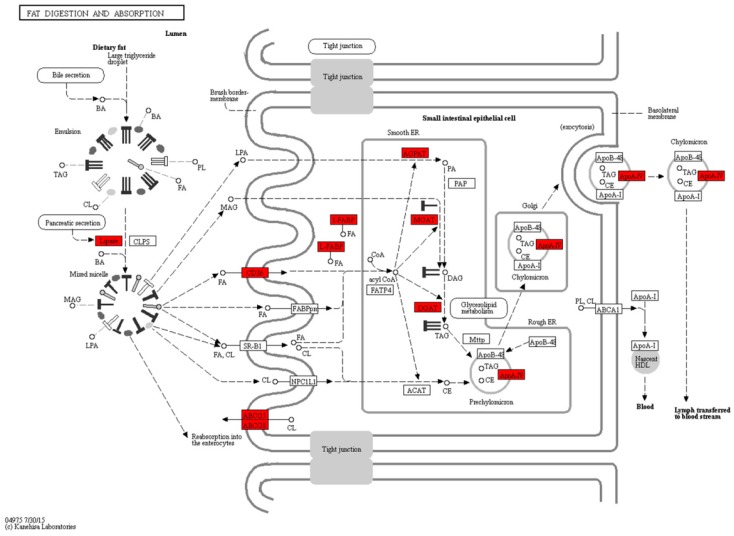
The differentially expressed genes involved in the fat digestion and absorption were annotated by KEGG. Red indicates up-regulated genes. The DEGs included *cel.1*, *cel.2*, *pla2g12a*, *pla2g1b*, *pla2g3*, *fabp2*, *fabp6*, *fabp1b.1*, *fabp10a*, *cd36*, *agpat2*, *mogat2*, *dgat1a*, *apoa4a*, *apoa4b.2*, and *apoa4b.3*.

Figure 5 shows that a large number of the DEGs involved in the fat digestion and absorption pathway were annotated by the KEGG pathway enrichment analysis during the mouth-opening stage. In the process of exogenous lipid digestion, five DEGs (*cel.1*, *cel.2*, *pla2g12a*, *pla2g1b*, and *pla2g3*) belonged to lipases and showed different up-regulation profiles. Similar results were found in the free fatty acid transportation and triglyceride re-synthesis processes, with four DEGs (*fabp2*, *fabp6*, *fabp1b.1*, and *fabp10a*) encoding fatty acid binding proteins and three DEGs (*agpat2*, *mogat2*, and *dgat1a*) encoding acyltransferase. Furthermore, three apolipoproteins (*apoa4a*, *apoa4b.2*, and *apoa4b.3*) involved in the transport of lipids were significantly up-regulated. In the fat digestion and absorption pathway, all 16 DEGs showed up-regulated profiles during the mouth-opening stage.

The absorption of exogenous fatty acids are either used for lipid storage or energy supply. In the present study, the up-regulated profile of *cd36* was observed in the switch from endogenous to exogenous feeding (Table S3), and it is a marker for adipogenesis in mammalian models. Conversely, we observed a significantly decreased expression profile for the key genes involved in fatty acid metabolism, including carnitine-palmitoyl transferase transporter isoforms *cpt1aa*, *cpt1ab*, and *cpt2*. Moreover, besides of the three isoforms, *acads*, *acat2*, *eci1*, and *aldh9a1b* also were identified that showed down-regulated profiles in the fatty acid metabolism pathway, whereas only *acsl5*, *acox1*, and *acox3* showed up-regulated profiles (Table S3). Obviously, the number of DEGs with down-regulated profiles in fatty acid metabolism was higher than the number with up-regulated profiles.

**Figure 6:**
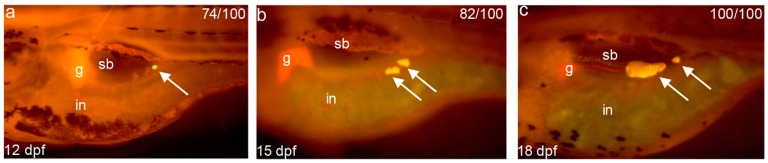
Nile Red staining reveals adipogenesis in zebrafish larval phase. Live zebrafish was stained with Nile Red at: 12 dpf (**a**); 15 dpf (**b**) and 18 dpf (**c**). White arrows annotate adipocyte neutral lipid droplets. The rate of adipogenesis shows on the top right corner. Swim bladder (sb), gall bladder (g), and intestine (in) are indicated. Bars = 400 μm in (**a**–**c**).

The above gene expression profiles revealed that during the mouth-opening stage, zebrafish larvae show increased lipid deposition and reduced fatty acid metabolism; therefore, we labeled neutral lipid droplets with Nile Red to explore adipogenesis in zebrafish larvae after mouth-opening feeding. The results showed that, under normal feeding condition, significant neutral lipid droplets were firstly observed commencing at 12 dpf and approximately 74% was labeled (Figure 6a), and this value increased in number and distribution with the growth of zebrafish (Figure 6b,c).

### 2.5. Temporal Expression of the Growth Hormone and Insulin-Like Growth Factor-I (GH-IGF-I) System and Growth Pattern during the Mouth-Opening Stage

In the present study, several factors of the growth hormone (GH)-insulin-like growth factor-I (IGF-I) system were identified. Growth hormone 1 (*gh1*) is a protein that stimulates growth and cell reproduction in most animals, and it presented an up-regulated expression profile from ENDO to EXO; however, other molecules associated with growth hormone, such as growth hormone releasing hormone (*ghrh*), growth hormone releasing hormone receptor (*ghrhr1*, *ghrhr2*, *ghrhra*, and *ghrhrb*), growth factor receptor-bound protein 2 (*grb2a* and *grb2b*), and GRB2-associated binding protein (*gab1* and *gab2*), displayed no significant difference. Moreover, insulin-like growth factors (IGFs) and insulin-like growth factors receptors (IGFRs) did not show significant differences. However, IGFBP1, which is present as two isoforms, *igfbp1a* and *igfbp1b*, displayed similar up-regulated profiles with development. A completely inverse expression profile was observed for *igfbp2a*. Other IGFBPs, including *igfbp2b*, *igfbp3*, *igfbp5*, and *igfbp6*, did not display differences in expression during the mouth-open stage (Table S3).

**Figure 7:**
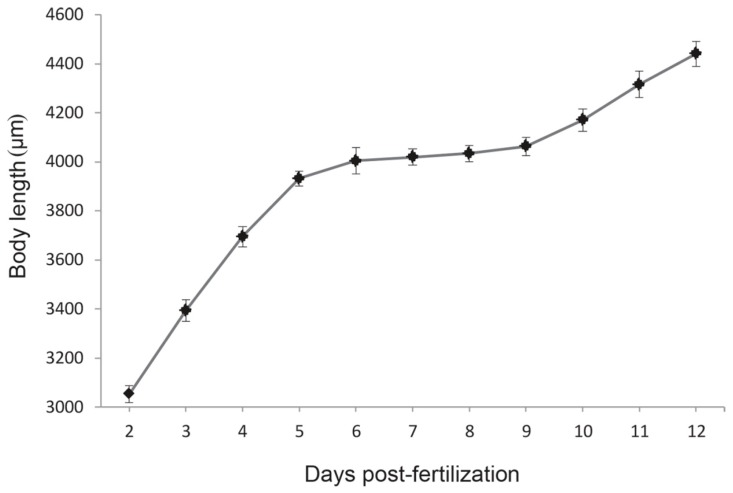
The drawing of growth trends from 2 days post fertilization (dpf) to 12 dpf. Data from body length are shown as mean ± standard deviation (*n* = 30).

**Figure 8:**
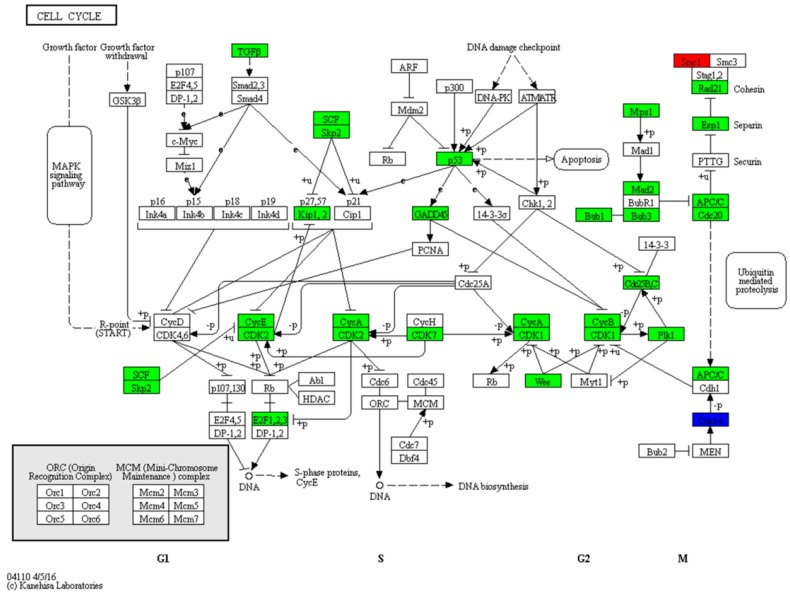
The differentially expressed genes associated with the cell cycle were annotated by KEGG. Red indicates up-regulated genes, green indicates down-regulated genes, and blue indicates genes that were both up- and down-regulated. The DEGs included *rbx1*, *skp2*, *tgfb2*, *cdkn1ca*, *ccna2*, *ccnb3*, *ccne2*, *cdk2*, *e2f2*, *e2f3*, *tp53*, *gadd45ga*, *cdk7*, *cdk1*, *wee1*, *ccnb1*, *ccnb2*, *ttk*, *mad2l1*, *bub1*, *bub3*, *cdc25b*, *plk1*, *smc1a*, *rad21a*, *espl1*, *cdc20*, *cdc23*, *anapc10*, *anapc13*, *cdc14aa*, *cdc14b*. “+p” indicates phosphorylation, “−p” indicates dephosphorylation, “+u” indicates ubiquitination, and “T-bar” indicates inhibition.

To further explore the growth pattern in zebrafish larvae during the mouth-opening stage, we measured the body length of the larvae and drew a growth curve from hatching to the exogenous feeding stage (Figure 7). The fastest increase in body length was observed at the endogenous feeding stage (2–5 dpf), which was followed by a significant decrease to almost growth arrest at the mixed feeding stage (5–8 dpf), and then a high increase in growth was gradually restored at 9 dpf. This phenotype was consistent with the global gene expression profiles of the cell cycle pathway. The RNA-seq analysis revealed that 32 DEGs were annotated in the cell cycle pathway (Figure 8), including at the G1, S, G2, and M phases. Among the multiple steps during cell division, significantly down-regulated profiles were observed for 30 out of the 32 functional genes, including genes for cyclin-dependent kinases (*cdk1*, *cdk2*, and *cdk7*), cyclins (*ccna2*, *ccnb1*, *ccnb2*, *ccnb3*, and *ccne2*), polo-like kinase 1 (*plk1*), cell division cycle 25B (*cdc25b*), and mitotic checkpoint serine/threonine kinase (*bub1*, *bub3*). Only one gene encoding the structural maintenance of chromosomes 1A (*smc1a*) and one gene encoding the cell division cycle 14Aa (*cdc14aa*) showed up-regulated profiles.

### 2.6. Expression of the Antigen Processing and Presentation Pathway during the Mouth-Opening Stage

**Figure 9:**
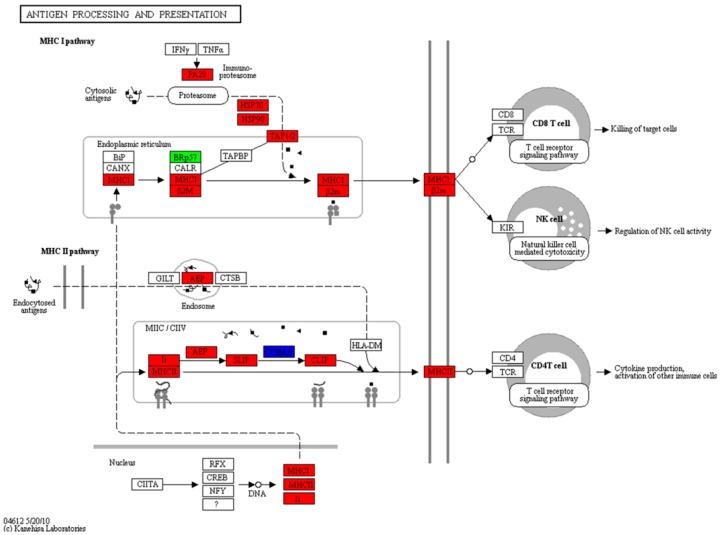
Significantly differentially expressed genes identified by KEGG as involved in the antigen processing and presentation pathway. Red indicates up-regulated genes, green indicates down-regulated genes, and blue indicates genes that were both up- and down-regulated. The DEGs included *psme1*, *psme2*, *psme3*, *hsc70*, *hsp90aa1.2*, *tap1*, *b2m*, *b2ml*, *mhc1zea*, *cd74a*, *cd74b*, *lgmn*, *ctssb*, *mhc2dab*, *si:busm1-266f07.2*, *si:busm1-194e12.12*, *ctssb.2*, *zgc: 100906*, *ctssb.1*, *and ctsla*.

The RNA-seq analysis revealed that a significant number of genes associated with protein degradation and transportation in the antigen processing and presentation pathway were differentially expressed in zebrafish larvae during the mouth-opening stage (Figure 9). Significantly up-regulated gene profiles were observed in the major histocompatibility complex (MHC)-I pathway, including the proteasome activator (*psme1* and *psme2*), heat shock protein superfamilies (*hsc70* and *hsp90aa1.2*), transporter of antigen presentation (*tap1*), beta (β)-2-microglobulin (*b2m* and *b2ml*) and MHC class I ZEA protein (*mhc1zea*) genes. A similar change in expression was also observed in the genes related to the MHC-II pathway, such as the CD74 molecule, MHC, class II invariant chain (*cd74a* and *cd74b*), legumain (*lgmn*), cathepsin Bb (*ctssb*), MHC class II DAB gene (*mhc2dab*), MHC class II alpha (α) chain (*si:busm1-266f07.2*), MHC class II β chain (*si:busm1-194e12.12*), and cathepsin S,b.2 (*ctssb.2*) genes. In the present study, the entire antigen processing and presentation pathway, which includes nine genes involved in the MHC-I pathway and eight genes involved in the MHC-II pathway, showed up-regulated profiles. However, only one gene encoding disulfide-isomerase A3 precursor (*zgc: 100906*) in the MHC-1 pathway and two genes encoding cathepsin S,b.1 (*ctssb.1*) and cathepsin La (*ctsla*) in the MHC-II pathway showed down-regulated profiles.

### 2.7. qPCR Analysis of Genes in the Antigen Processing and Presentation Pathway

**Figure 10:**
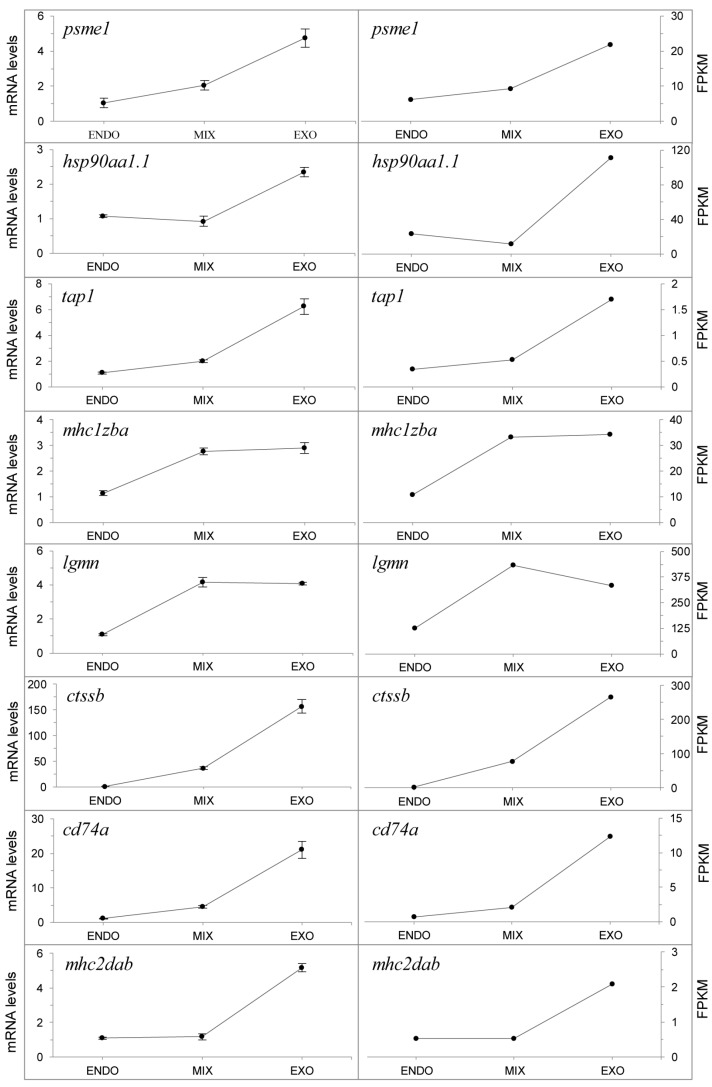
Gene expression levels were revealed by: qPCR (**left**) and RNA-seq (**right**). The proteasome activator (*psme1*), heat shock protein 90 kDa α (*hsp90aa1.1*), antigen transporter (*tap1*), MHC class I ZBA (*mhc1zba*), legumain (*lgmn*), cathepsin Bb (*ctssb*), CD74 molecule (*cd74a*), and MHC class II DAB (*mhc2dab*) were validated. Data from qPCR are shown as mean ± standard deviation (*n* = 3). FPKM from RNA-seq are means of two replicates.

**Figure 11:**
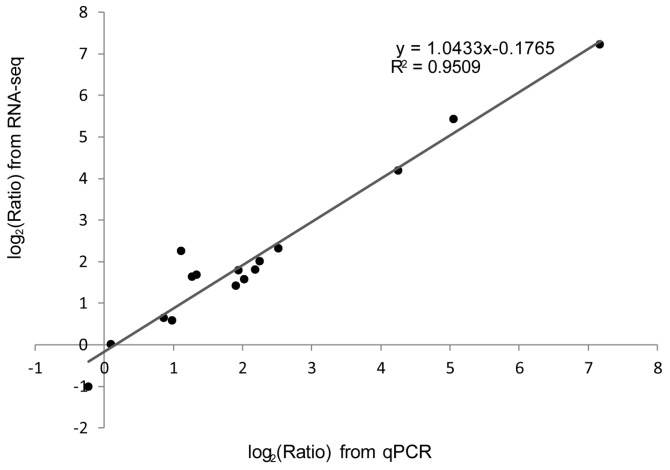
Correlation analysis of fold change data between qPCR and RNA-seq. Scatterplots were generated by the log_2_ expression ratios from RNA-seq (*x*-axis) and qPCR (*y*-axis). Data from qPCR are means of three replicates, while data from RNA-seq are means of two replicates. The reference line represents the linear relationship between the results of RNA-seq and qPCR.

To validate the reproducibility and accuracy of the RNA-seq analysis results, we determined the relative mRNA levels for eight genes in the antigen processing and presentation pathway via qPCR. Primers of the candidate genes are shown in Table S5. The results showed that the qPCR expression patterns for candidate genes were consistent with those of the RNA-seq data (Figure 10). Furthermore, the fold change of the gene expression ratios between the RNA-seq and qPCR analyses were significantly positive correlated (*R*^2^ = 0.9509) as revealed by a linear regression analysis (Figure 11). This finding indicated the reliability of the RNA-seq data.

**Figure 12:**
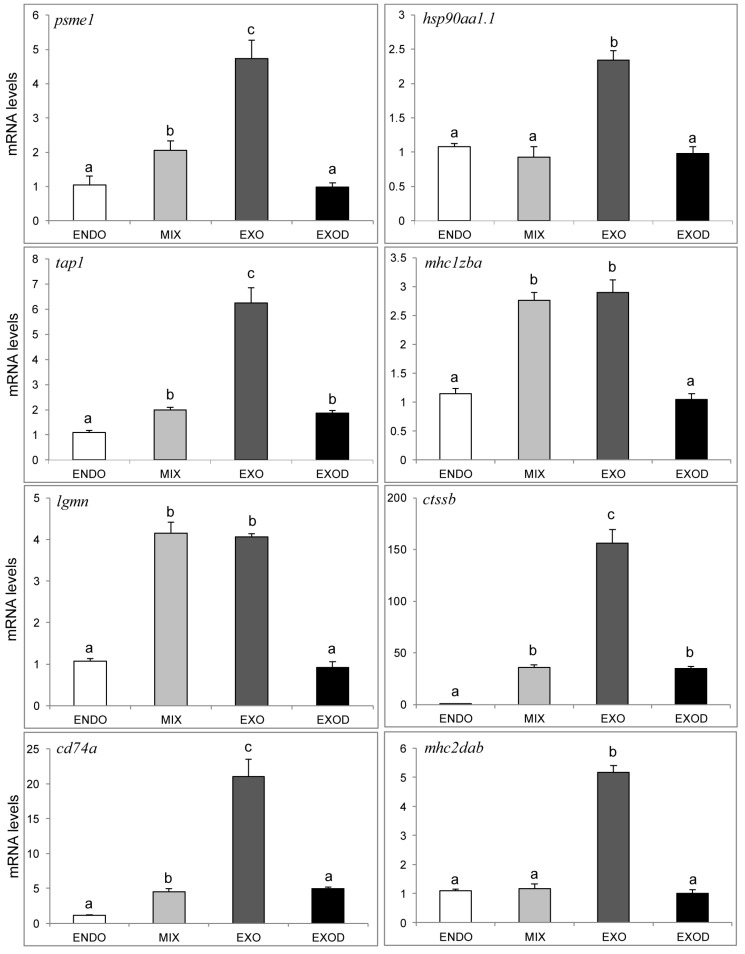
qPCR analysis of the genes related to antigen processing and presentation, including ENDO, MIX, EXO and EXOD groups. The proteasome activator (*psme1*), heat shock protein 90 kDa α (*hsp90aa1.1*), antigen transporter (*tap1*), MHC class I ZBA (*mhc1zba*), legumain (*lgmn*), cathepsin Bb (*ctssb*), CD74 molecule (*cd74a*), and MHC class II DAB (*mhc2dab*) were detected. Data from qPCR are shown as mean ± standard deviation (*n* = 3). Significant differences are marked by different letters (*p* < 0.05).

Next, to determine whether the activation of the MHC pathways during the mouth-opening stage requires a supply of exogenous nutrients, the same gene expression levels were determined at the exogenous feeding phase without exogenous nutrition (EXOD, 8 dpf) via qPCR. The results showed that eight genes involved in antigen processing and presentation were significantly down-regulated in the EXOD group. The *psme1*, *hsp90aa1.1*, *mhc1zba*, *lgmn* and *mhc2dab* fell to ENDO group expression level, and the *tap1*, *ctssb*, *cd74a* fell to MIX group level (Figure 12). The results suggested that the activation of the MHC pathways were highly dependent on the supply of exogenous nutrients at the mouth-opening stage.

## 3. Discussion

Fish larvae are vulnerable and present high mortality during the mouth-opening stage because of their physiological characteristics. Limited data are available on the basic biology of this phase, which has severely hampered the development of the aquaculture industry. To date, several studies have performed transcriptomic analyses and reported the physiological, biochemical and behavioral processes associated with larval development in oviparous fish species, such as *Sparus aurata* [22], *Gadus morhua* L. [23], *Gymnocypris przewalskii* [24], *Paralichthys olivaceus* [25], and *Dicentrarchus labrax* [26]. However, few studies have performed transcriptome analyses of the mouth-opening stage. In the present study, the RNA-seq approach was used to collect data on the transition of physiological processes during the mouth-opening stage and investigate the gene expression patterns in zebrafish larvae. We identified 4965 DEGs in the 26,593 genes expressed in the zebrafish larvae, and they included 2172 genes with up-regulated profiles and 2313 with down-regulated profiles. The DEGs involved in biological processes and pathways, including the arachidonic acid metabolism, linoleic acid metabolism, fat digestion and absorption, and antigen processing and presentation pathways, were activated, whereas DEGs involved in the cell cycle, homologous recombination, and fatty acid metabolism pathways were inhibited. These data provide insights into the molecular mechanisms underlying the larval mouth-opening stage and could be useful for aquaculture development research.

### 3.1. Exogenous Fatty Acids Are Used for Lipid Deposition Instead of Metabolism during the Mouth-Opening Stage

Exogenous feeding is essential for the survival of fish larvae, especially after yolk-sac resorption [1]. By utilizing yolk-sac reserves, digestive organs, such as the liver, pancreas and intestines, gradually develop to maturation. The specific enzymes involved in energy storage or consumption are activated in the organs involved in the digestive tract [16]. In the present study, RNA-seq analyses revealed that a large number of DEGs of the fat digestion and absorption pathway were enriched during the mouth-opening stage. In the process of fat digestion, DEGs for digestive enzymes that contain various lipases and phospholipases, such as *cel.1*, *cel.2*, *pla2g12a*, *pla2g1b* and *pla2g3*, displayed strongly up-regulated profiles. Digestive enzymes primarily hydrolyze dietary fats to release free fatty acids, monoglycerides, lysophosphatidic acids, and cholesterol to the intestine [27]. Similar expression profiles were observed for several DEGs for fatty acid binding protein (FABP)s, such as *fabp1b.1* [28], *fabp2* [29], and *fabp6* [30], which were mainly expressed in the intestine, and *fabp10a* [31], which is expressed in the liver. This observation is consistent with the previous study [32] that the uptake of exogenous fatty acids requires the mobilization of various FABPs. Moreover, several key genes involved in the re-synthesis of triglycerides (TAG) from the digestion products of dietary fats, including *agpat2*, *mogat2*, and *dgat1a*, displayed significantly up-regulated profiles. Currently, *agpat2*^-/-^, *mogat*^-/-^, and *dgat1*^-/-^ mouse models are well established [33,34,35]. Although the deletion of these three genes was not lethal, fat absorption was decreased significantly. Apolipoprotein binds with lipids to form lipoproteins for the transportation of lipids to various tissues [36]. In our study, three of the four ApoA-IV genes, including *apoa4a*, *apoa4b.2*, and *apoa4b.3*, showed that up-regulated profiles. The result is consistent with the results of a previous study showed that ApoA-IV performs a conserved role involved in regulating food intake in mammals and teleost fishes [37]. Together, the up-regulation of these genes revealed a successful transition for the conversion of exogenous to endogenous fat during the mouth-opening stage.

Further, we identified a significant negative correlation in the gene expression profiles of molecular markers between lipid deposition and fatty acid metabolism, including the *cd36*, and *cpt1aa*, *cpt1ab*, and *cpt2*, respectively. Several studies have revealed that during the process of lipid deposition, *cd36* has a rate-limiting function in triglyceride synthesis in muscle and adipocytes [38,39,40]. In our study, a significantly up-regulated expression profile of *cd36* was observed during the mouth-opening stage. This molecular signature indicated that the uptake of exogenous fatty acids obviously promoted lipid deposition. The genes *cpt1* and *cpt2* represent molecular markers and were involved in the oxidation of free fatty acids in mitochondria [41]. However, the significant down-regulation of *cpt1aa* and *cpt1ab* and the *cpt2* isoform indicated that increases in lipid deposition during the mouth-opening feeding stage were accompanied by decreases in fatty acid metabolism. This phenotype was also observed in the alevin stage of trout [2], suggesting that the physiological switch for lipid deposition at the mouth-opening stage may be a common feature in oviparous fish. This result was further corroborated by Nile Red staining of neutral lipid droplets, which showed increased accumulation in the visceral adipocytes after exogenous feeding. In adult fish, the endogenous reserves of lipids meet the metabolic demands of the fish during fasting [42,43] and supply energy for exercise [44] and cold acclimation [45]; thus, these reserves are essential for the survival of fish throughout their life cycle. Our data verified that lipid deposition in zebrafish larvae commences with the mouth-opening stage.

### 3.2. Zebrafish Larvae Growth Is Retarded by the Cell Cycle Pathway during the Mouth-Opening Stage

The GH/IGF-I system is important for the regulation of growth and development in fish [46]. Previous studies revealed that the key components of the GH/IGF-I axis, which includes GH, GHRH, IGF-I, IGF-1R, and IGFBP, showed different mRNA expression level patterns during the larval stages [15,47], and studies have identified the turning point in expression patterns occurred at the mouth-opening stage. For example, in the common sole, GHRH and GH mRNA expression is significantly up-regulated soon after mouth opening and then decreases until the juvenile stage. Moreover, IGF-I, IGFR1, and IGFBP1 mRNA displays lower expression before mouth opening and is up-regulated after the first meal [15]. These switches in gene expression profiles during the mouth-opening stage indicate where essential adjustments for growth and development occur. In the present study, we observed that *gh1* was up-regulated, which is consistent with previous findings in common sole [15]. However, other molecular markers associated with growth hormones did not display significant differences at this stage. We further observed that the mRNA of the *igfbp1a* and *igfbp1b* isoforms was up-regulated and the growth rate in body length was decreased during the mouth-opening stage. This observation is consistent with previous studies conducted in zebrafish in which increases in IGFBP1 mRNA expression caused growth and developmental retardation [48].

Furthermore, the RNA-seq analyses revealed that the inhibition of body length growth was mainly associated with the regulation of the cell cycle pathway because a dramatic down-regulation of the genes encoding for associated cell division proteins was observed. Cell division is an energy-consuming process in which the amount of DNA in a cell is doubled in the S phase [49] and protein is synthesized in the G_2_ phase [50]. Cell cycle arrest may be one of the most efficient methods for reducing energy utilization. In the present study, saturated feeding observed cell cycle arrest, suggested that the reduced growth rate during the mouth-opening stage is a physiological switch and ontogenetic process in zebrafish larvae. This phenomenon supports the hypothesis that most of the energy accumulated in the mouth-opening stage tends to be reserved or used for specific demands, such as immune system development or lipid deposition, instead of rapid growth.

### 3.3. Activation of the MHC Pathway Depends on the Supply of Exogenous Nutrients during the Mouth-Opening Stage

To date, most studies on the teleost immune system in adult fish have focused on the head, kidney, spleen, and liver [51,52,53,54]. Findings have shown that the MHC class of molecules involved in the antigen processing and presentation plays an important role in these tissues. MHC class I molecules mainly survey the endogenous antigens of the cytosol, where they are degraded into proteasomal products and then transported to CD8+ T cell surfaces, where they stimulate the cells to mature into effectors [55]. However, MHC class II molecules present exogenous antigens to the immune system for the recognition and activation of CD4+ T cells via the endocytic route [56]. Although the role of the MHC pathways in immune recognition and disease resistance has been extensively studied in vertebrates [57,58], information is lacking on the MHC pathways associated with genes expression profile during larval development at the mouth-opening stage. After mouth opening, a variety of microorganisms enter the gastrointestinal tract and microflora colonization begins immediately; therefore, we hypothesized that the larvae would be more susceptible to pathogen infection through the gastrointestinal tract and the coordinated expression of the MHC during the mouth-opening stage may participated in antigen recognition. In the present study, we found that a significant number of DEGs involved in antigen processing and presentation in the MHC class I and II pathways showed significantly up-regulated profiles. This result confirmed our hypothesis that microflora colonization at the mouth-opening stage activates a strong antigen recognition pathway.

Next, we studied the correlation between exogenous nutrition and MHC pathways. The mouth-opening stage is extremely susceptible to hunger stress, and the importance of nutrition is widely recognized in regulating the ontogenesis, development, intensity and type of immune response [59]. To explore whether nutritional supply was required in the activation of the MHC pathways during the mouth-opening stage, we detected the expression levels of eight genes in the MHC pathways by qPCR at the exogenous feeding phase without exogenous nutrition. The result showed eight genes were significantly attenuated in the absence of exogenous nutrition, indicating that exogenous nutrition is an important activator of the MHC pathway. Furthermore, we compared the DEGs involved in the MHC pathways identified in our study with those in the response to long term starvation in the adult large yellow croaker (*Larimichthys crocea*) [42]. The inconsistent result was observed that the latter not alter the gene expression level associated with MHCs pathway under starvation (Table S6). Studies have revealed that adipose tissue is closely related to immune functions and part of the immune system [60], and fasting led to a significant activation involved in fatty acid metabolism pathway. Changes were not observed in the MHC pathways in response to starvation in adult large yellow croaker, indicating the importance of endogenous lipids in maintaining the immune recognition level. In the present study, the utilization of exogenous fatty acids during the mouth-opening stage showed a tendency for lipid deposition rather than metabolism and may be involved in subsequent immune defense.

## 4. Materials and Methods

### 4.1. Larvae Collection and RNA Extraction

The adult fish were reared and the embryos were incubated in accordance with standard protocols [61]. The temperature (28.5 °C) and 14 h light/10 h dark cycle were strictly controlled in the zebrafish circulation aquaculture system. After fertilization, the embryos were immediately collected and incubated in 100 mm diameter culture dishes (60 larvae per dish). At 105 hpf, the larvae were transferred into tanks containing 1 liter of fresh egg water, and the Larval AP100 diet (Haisheng, Shanghai, China) was supplied twice per day. At specific developmental stages, including the endogenous (96 hpf), mix (120 hpf) and exogenous (192 hpf) feeding stages, the larvae were sacrificed using the anesthetic tricaine, and RNA was extracted for the RNA-seq analysis. Meanwhile, the same samples were stored at −80 °C until RNA extraction for the real-time qPCR analysis. Animal experiments were conducted in accordance with the regulations of the Guide for Care and Use of Laboratory Animals, and they were approved by the Committee of Laboratory Animal Experimentation at Southwest University.

### 4.2. cDNA Library Construction and Illumina Deep Sequencing

Two independent biological replicates of each phase, including 96, 120, and 192 hpf, were used for library construction and RNA-seq, and a single biological replicate are 60 fish larvae. The whole fish larvae were subjected to RNA extraction (Takara, Dalian, Japan) according to the manufacturer’s protocol. For each sample, the quality of total RNA was examined via agarose gel electrophoresis, and the total RNA concentration was measured using a SmartSpecTMPlus spectrophotometer (Bio-Rad, Hercules, CA, USA). The RNA libraries were constructed by Gene Denovo Co. (Guangzhou, China). RNA integrity and quality were measured with an Agilent 2100 Bioanalyzer (Agilent Technologies, Palo Alto, CA, USA), and the RNA integrity number (RIN) index was calculated for each sample. Only RNA with a RIN number > 8.5 were processed further. Total mRNA was used for isolation with oligo (dT) magnetic bead enrichment, and small segments fragmented with fragment buffer were added and reverse transcribed with 6 bp random primers. Double-strand cDNA was synthesized using buffer, dNTPs, DNA polymerase I and RNase H. After purifying the double-strand cDNA fragments using a QiaQuick PCR extraction kit, the cDNA was subjected to end repair, dA-tailing and adapter ligation. Agarose gel electrophoresis was used to collect the processing and PCR amplification products. The amplified fragments were sequenced using an Illumina HiSeq 2000 system. The sequencing data were deposited in the NCBI Sequence Read Archive under the accession number SRR4045953.

### 4.3. Bioinformatic Analysis of RNA-Seq Data

To acquire clean reads, the raw reads were filtered by removing the adapter-containing reads and reads with more than 10% unknown nucleotides and low-quality reads (Q < 20). To avoid interference of ribosomal RNA, we mapped the clean reads to the ribosome database using *bowtie* [62], and the matched reads were removed from the ribosome database. The remaining reads were mapped to the genome sequence of zebrafish (GRCz10) using *TopHat* [63]. The aligned reads were assembled into transcripts using *cufflinks*, and the assembled transcripts were merged using *cuffmerge*. The abundance of gene transcripts was calculated via FPKM (Fragments per kilobase of transcript per million mapped reads) [64,65], and genes with a mean abundance >0 FPKM in any one of these samples were regarded as being expressed.

The raw read counts were used to differential expression analysis using *egdeR* [66]. Genes with fold change of |log_2_FC| > 1, *p* < 0.05 and FDR < 0.05 were considered differentially expressed. In this study, the DEGs between the ENDO and MIX, ENDO and EXO, and MIX and EXO groups were analyzed. Moreover, an expression trend analysis was performed with ENDO as the control group and MIX or EXO as the treatments, and the gene expression levels were normalized to 0, log_2_ (υMIX/υENDO), and log_2_ (υEXO/υENDO). DEGs were clustered using STEM [67], which is used to cluster and compare short time series gene expression data with its visualization capabilities and study dynamical biological processes. The clustered profiles at *p* < 0.05 were regarded as significant DEGs.

After obtaining the DEGs, a gene ontology (GO) classification of the DEGs was performed using WEGO [68], and the terms included molecular function, cellular component and biological process. The KEGG pathway was annotated by the KEGG Automatic Annotation Server (KAAS). The hypergeometric test was used to identify overrepresented GO and KEGG pathway terms with a significance level (*p* < 0.05) and Beniamini and Hochberg method was used for the correction of the *p*-values.

### 4.4. Zebrafish Larvae Adipocytes Staining and Measure of Body Length

Adipocytes were stained and measured in whole zebrafish using the Nile Red method [69]. Zebrafish larvae were placed in dishes (40 larvae per dish) and stained with Nile Red (final working concentration 1.0 μg/mL) for 15 min at 28 °C in the dark. After anesthetizing using Tricaine (Sigma-Aldrich, St. Louis, MO, USA), the zebrafish larvae were mounted on a microslide and imaged using a Leica DM6000B fluorescence stereomicroscope (Leica, Wetzlar, Germany). The body length (distance between the tip of the snout and the end of the caudal vertebra) was measured, and the values were presented as the mean ± standard deviation (*n* = 20).

### 4.5. qPCR Analysis

A qPCR analysis was performed using the CFX96 Real-Time PCR Detection System (Bio-Rad, Hercules, CA, USA) with SYBR Green (Promega, Madison, WI, USA) as the fluorescent detection dye according to the manufacturer’s protocol. Using oligo(dT) as a primer, first-strand cDNA for each sample was synthesized from approximately 2 μg of total RNA and applied as a template for the qPCR assay with gene-specific primers. The Primer Premier 5.0 software was used to design the specific primers. The RNA-seq analysis showed that *actb2* was not differentially expressed among the samples and showed high expression levels; therefore, it was used as an internal reference for the normalization of gene expression. The amplification was conducted in a volume of 20 μL containing 10 μL of SYBR Green PCR Master Mix. The qPCR amplification protocol was as follows: 95 °C for 3 min, followed by 40 cycles of 95 °C for 15 s, 60 °C for 20 s, and 72 °C for 20 s. The melting curve for PCR products was generated by heating from 65 to 95 °C in 0.5 °C increments and a 5 s dwell time, and a plate read was performed at each temperature. The specificity of the PCR fragments was detected by analyzing the melting curve and agarose gel electrophoresis results. Three biological replicates for each sample were performed. The expression levels of the genes were determined by the 2^−ΔΔ*C*t^ method [70].

## 5. Conclusions

The present study revealed the gene expression profiles underlying the transition from endogenous to exogenous feeding in zebrafish larvae. Changes in several pathways and molecular markers revealed a physiological process that promotes energy storage during the mouth-opening stage via increased lipid storage, decreased fatty acid metabolism, and arrested cell division. Moreover, the MHC-I and MHC-II processing pathways were significantly enhanced in these stages and the activation of these MHC pathways requires a supply of exogenous nutrients. These findings will facilitate further investigations of the molecular mechanisms underlying the physiological changes that occur during the transition from endogenous to exogenous feeding and should be beneficial for the aquaculture industry.

## Figures and Tables

**Table 1:** Statistics for the filtering and mapping of reads.

Libraries	ENDO-1	ENDO-2	MIX-1	MIX-2	EXO-1	EXO-2
Raw reads (M)	21.19	24.05	22.37	24.03	26.06	25.28
Clean reads (M)	20.74	23.54	21.84	23.47	25.50	24.71
Percent clean reads	97.88	97.89	97.62	97.69	97.85	97.73
Processed reads	20.37	23.05	21.16	22.74	24.91	23.97
Mapped reads (M)	17.33	19.14	16.97	18.43	20.80	19.45
Percent mapped	85.07	83.06	80.20	81.05	83.50	81.12
Unique mapping (M)	16.96	18.81	16.67	18.09	20.41	19.12
Percent uniquely mapped reads ^a^	97.86	98.28	98.23	98.16	98.13	98.30

^a^ The percent uniquely mapped reads indicates the ratio of unique mapping to the mapped reads.

**Table 2:** Top 10 KEGG pathways with high representation of the DEGs.

Pathways	No. of DEGs with Pathway Annotation							Pathway Identification (ID)
	All Profiles	Profile 0	Profile 1	Profile 3	Profile 4	Profile 6	Profile 7	
Antigen processing and presentation	23	2	0	1	4	5	8	ko04612
Arachidonic acid metabolism	19	1	2	1	2	10	3	ko00590
Metabolic pathways	246	59	26	37	17	38	51	ko01100
Cell cycle	32	9	2	19	0	1	1	ko04110
Linoleic acid metabolism	10	0	0	1	3	4	2	ko00591
Homologous recombination	13	4	0	8	0	0	0	ko03440
Fat digestion and absorption	16	0	0	0	2	6	8	ko04975
Arginine and proline metabolism	24	4	1	3	3	5	5	ko00330
Glutathione metabolism	18	3	1	6	0	3	3	ko00480
α-Linolenic acid metabolism	8	0	0	0	1	2	5	ko00592

## Supplementary Materials

Supplementary materials can be found at www.mdpi.com/1422-0067/18/8/1634/s1.

## Abbreviations

α	Alpha
β	Beta
DEGs	Differentially expressed genes
dpf	Days post fertilization
ENDO	Endogenous feeding
EXO	Exogenous feeding
FABP	Fatty acid binding protein
FDR	False discovery rate
FPKM	Fragments per kilobase of transcript per million mapped reads
GO	Gene ontology
hpf	Hours post fertilization
ID	Identification
KEGG	Kyoto encyclopedia of genes and genomes
MHC	Major histocompatibility complex
MIX	Mixed feeding
PCA	Principal component analysis
PCs	Principal components
qPCR	Quantitative real-time polymerase chain reaction
RIN	RNA integrity number
SRA	Sequence read archive
STEM	Short time-series expression miner software
TAG	Triglycerides
WEGO	Web gene ontology annotation plot
