# Supplementary material for: Transcriptome Analysis Reveals Increases in Visceral Lipogenesis and Storage and Activation of the Antigen Processing and Presentation Pathway during the Mouth-Opening Stage in Zebrafish Larvae
Source: Int J Mol Sci. 2017 Jul 31;18(8):1634. doi: 10.3390/ijms18081634 (PMC5578024; doi:10.3390/ijms18081634)
Supplement: Supplementary file 1 [file ijms-18-01634-s001.zip › Table S6.pdf]

Table S6 Differentially expressed genes related to the antigen processing and presentation pathways

Gene name	Gene ID	Description	Fold -change	*p*-vaule
mhc1	CL4289.Contig2_All	major histocompatibility complex, class I	-1.1758	3.22E-92
mhc2	Unigene8098_All	MHC class II antigen alpha chain, class II	1.0466	5.83E-09
hsp27	CL4186.Contig2_All	Heat shock 27kDa protein	3.9285	2.09E-30
hsp70	Unigene6318_All	Heat shock 70kDa protein	3.7239	2.05E-11
b2m	CL695.Contig1_All	Beta-2-microglobulin	1.2273	3.22E-24
tap1	Unigene30122_All	Transporter of antigen presentation	1.6031	3.90E-06
ctsb	Unigene4878_All	Cathepsin b	-1.006	9.39E-82
ctse	Unigene1543_All	Cathepsin E	3.6175	3.22E-24
ctsf	Unigene26177_All	Cathepsin F	1.9778	2.66E-76

The data are from the research of Qian et al. 2015 that the large yellow croakers at 21 days after fasting. Identification of all differentially expressed genes was based on $P < 0.05$. A P value < 0.05 indicated that the gene was significantly altered in fasting fish relative to that observed in normal feeding fish. The absolute value of “Fold-change” is the magnitude of up- or downregulation for each gene after fasting. “+” indicates upregulation and “-” indicates downregulation.

Qian, B.; Xue, L.; Huang, H. Liver transcriptome analysis of the Large yellow croaker (*Larimichthys crocea*) during fasting by using RNA-seq. *PLoS ONE* **2016**, *11*, doi:10.1371/journal.pone.0150240.
