# Supplementary material for: Transcriptome Analysis Reveals Increases in Visceral Lipogenesis and Storage and Activation of the Antigen Processing and Presentation Pathway during the Mouth-Opening Stage in Zebrafish Larvae
Source: Int J Mol Sci. 2017 Jul 31;18(8):1634. doi: 10.3390/ijms18081634 (PMC5578024; doi:10.3390/ijms18081634)
Supplement: Supplementary file 1 [file ijms-18-01634-s001.zip › Table S1.pdf]

Table S1 GO-BP enrichment analysis for the non-detected genes during the mouth-opening stage

Gene Ontology term	Cluster frequency	Genome frequency of use	P-value	Corrected P-value
neurological system process	88 out of 250 genes, 35.2%	318 out of 10793 genes, 2.9%	1.96E-73	9.23E-71
system process	90 out of 250 genes, 36.0%	444 out of 10793 genes, 4.1%	3.7E-62	1.73E-59
sensory perception of chemical stimulus	53 out of 250 genes, 21.2%	149 out of 10793 genes, 1.4%	1E-49	4.7E-47
sensory perception of smell	42 out of 250 genes, 16.8%	97 out of 10793 genes, 0.9%	1.1E-43	5.2E-41
sensory perception	53 out of 250 genes, 21.2%	212 out of 10793 genes, 2.0%	1.31E-40	6.18E-38
detection of chemical stimulus involved in sensory perception	36 out of 250 genes, 14.4%	84 out of 10793 genes, 0.8%	2.85E-37	1.33E-34
detection of chemical stimulus	36 out of 250 genes, 14.4%	87 out of 10793 genes, 0.8%	1.37E-36	6.42E-34
G-protein coupled receptor signaling pathway	65 out of 250 genes, 26.0%	436 out of 10793 genes, 4.0%	3.06E-35	1.43E-32
detection of stimulus involved in sensory perception	36 out of 250 genes, 14.4%	98 out of 10793 genes, 0.9%	2.43E-34	1.14E-31
detection of stimulus	37 out of 250 genes, 14.8%	119 out of 10793 genes, 1.1%	3.47E-32	1.63E-29
signal transduction	124 out of 250 genes, 49.6%	1890 out of 10793 genes, 17.5%	5.97E-32	2.8E-29
single organism signaling	125 out of 250 genes, 50.0%	1997 out of 10793 genes, 18.5%	3.29E-30	1.54E-27
signaling	125 out of 250 genes, 50.0%	1999 out of 10793 genes, 18.5%	3.64E-30	1.7E-27
response to stimulus	146 out of 250 genes, 58.4%	2688 out of 10793 genes, 24.9%	4.9E-30	2.29E-27
cell communication	125 out of 250 genes, 50.0%	2062 out of 10793 genes, 19.1%	8.15E-29	3.82E-26
cellular response to stimulus	125 out of 250 genes, 50.0%	2180 out of 10793 genes, 20.2%	1.94E-26	9.13E-24
cell surface receptor signaling pathway	67 out of 250 genes, 26.8%	764 out of 10793 genes, 7.1%	2.03E-22	9.55E-20
regulation of cellular process	139 out of 250 genes, 55.6%	3216 out of 10793 genes, 29.8%	7.98E-18	3.74E-15
biological regulation	148 out of 250 genes, 59.2%	3578 out of 10793 genes, 33.2%	1.24E-17	5.85E-15
regulation of biological process	139 out of 250 genes, 55.6%	3303 out of 10793 genes, 30.6%	9.92E-17	4.65E-14
single-multicellular organism process	98 out of 250 genes, 39.2%	1991 out of 10793 genes, 18.4%	6.78E-15	3.18E-12
multicellular organismal process	98 out of 250 genes, 39.2%	2005 out of 10793 genes, 18.6%	1.08E-14	5.06E-12
cellular process	200 out of 250 genes, 80.0%	6497 out of 10793 genes, 60.2%	1.07E-11	5.02E-09
response to chemical	40 out of 250 genes, 16.0%	529 out of 10793 genes, 4.9%	2.48E-11	1.16E-08
positive regulation of adenylate cyclase activity	10 out of 250 genes, 4.0%	32 out of 10793 genes, 0.3%	1.53E-09	0.000000718
positive regulation of cAMP metabolic process	10 out of 250 genes, 4.0%	33 out of 10793 genes, 0.3%	2.15E-09	0.000001
positive regulation of cAMP biosynthetic process	10 out of 250 genes, 4.0%	33 out of 10793 genes, 0.3%	2.15E-09	0.000001
regulation of adenylate cyclase activity	10 out of 250 genes, 4.0%	37 out of 10793 genes, 0.3%	7.46E-09	0.0000035
regulation of cAMP metabolic process	10 out of 250 genes, 4.0%	38 out of 10793 genes, 0.4%	9.93E-09	0.00000465
regulation of cAMP biosynthetic process	10 out of 250 genes, 4.0%	38 out of 10793 genes, 0.4%	9.93E-09	0.00000465
positive regulation of cyclase activity	10 out of 250 genes, 4.0%	38 out of 10793 genes, 0.4%	9.93E-09	0.00000465
positive regulation of lyase activity	10 out of 250 genes, 4.0%	38 out of 10793 genes, 0.4%	9.93E-09	0.00000465
positive regulation of cyclic nucleotide metabolic process	10 out of 250 genes, 4.0%	39 out of 10793 genes, 0.4%	1.3E-08	0.00000613
positive regulation of cyclic nucleotide biosynthetic process	10 out of 250 genes, 4.0%	39 out of 10793 genes, 0.4%	1.3E-08	0.00000613
positive regulation of nucleotide biosynthetic process	10 out of 250 genes, 4.0%	39 out of 10793 genes, 0.4%	1.3E-08	0.00000613
positive regulation of nucleotide metabolic process	10 out of 250 genes, 4.0%	39 out of 10793 genes, 0.4%	1.3E-08	0.00000613
positive regulation of purine nucleotide biosynthetic process	10 out of 250 genes, 4.0%	39 out of 10793 genes, 0.4%	1.3E-08	0.00000613
positive regulation of purine nucleotide metabolic process	10 out of 250 genes, 4.0%	39 out of 10793 genes, 0.4%	1.3E-08	0.00000613
regulation of cyclase activity	10 out of 250 genes, 4.0%	43 out of 10793 genes, 0.4%	3.63E-08	0.000017
regulation of lyase activity	10 out of 250 genes, 4.0%	43 out of 10793 genes, 0.4%	3.63E-08	0.000017
regulation of nucleotide metabolic process	10 out of 250 genes, 4.0%	44 out of 10793 genes, 0.4%	4.61E-08	0.0000216
regulation of cyclic nucleotide metabolic process	10 out of 250 genes, 4.0%	44 out of 10793 genes, 0.4%	4.61E-08	0.0000216

regulation of cyclic nucleotide biosynthetic process	10 out of 250 genes, 4.0%	44 out of 10793 genes, 0.4%	4.61E-08	0.0000216
regulation of nucleotide biosynthetic process	10 out of 250 genes, 4.0%	44 out of 10793 genes, 0.4%	4.61E-08	0.0000216
regulation of purine nucleotide biosynthetic process	10 out of 250 genes, 4.0%	44 out of 10793 genes, 0.4%	4.61E-08	0.0000216
regulation of purine nucleotide metabolic process	10 out of 250 genes, 4.0%	44 out of 10793 genes, 0.4%	4.61E-08	0.0000216
cellular protein modification process	54 out of 250 genes, 21.6%	1153 out of 10793 genes, 10.7%	2.8E-07	0.00013
protein modification process	54 out of 250 genes, 21.6%	1153 out of 10793 genes, 10.7%	2.8E-07	0.00013
defense response to bacterium	10 out of 250 genes, 4.0%	53 out of 10793 genes, 0.5%	3.01E-07	0.00014
macromolecule modification	54 out of 250 genes, 21.6%	1183 out of 10793 genes, 11.0%	6.39E-07	0.00029
immune response	17 out of 250 genes, 6.8%	178 out of 10793 genes, 1.6%	7.66E-07	0.00035
positive regulation of phosphorus metabolic process	12 out of 250 genes, 4.8%	96 out of 10793 genes, 0.9%	2.04E-06	0.00095
positive regulation of phosphate metabolic process	12 out of 250 genes, 4.8%	96 out of 10793 genes, 0.9%	2.04E-06	0.00095
defense response to other organism	10 out of 250 genes, 4.0%	65 out of 10793 genes, 0.6%	2.17E-06	0.00101
regulation of mitotic cell cycle	9 out of 250 genes, 3.6%	54 out of 10793 genes, 0.5%	3.55E-06	0.00166
protein autophosphorylation	9 out of 250 genes, 3.6%	54 out of 10793 genes, 0.5%	3.55E-06	0.00166
membrane disruption in other organism	7 out of 250 genes, 2.8%	30 out of 10793 genes, 0.3%	4.24E-06	0.00199
response to bacterium	11 out of 250 genes, 4.4%	87 out of 10793 genes, 0.8%	4.89E-06	0.00229
defense response to Gram-positive bacterium	7 out of 250 genes, 2.8%	32 out of 10793 genes, 0.3%	6.74E-06	0.00316
modification of morphology or physiology of other organism	7 out of 250 genes, 2.8%	34 out of 10793 genes, 0.3%	0.0000103	0.00486
immune system process	27 out of 250 genes, 10.8%	470 out of 10793 genes, 4.4%	0.000012	0.00562
response to biotic stimulus	12 out of 250 genes, 4.8%	124 out of 10793 genes, 1.1%	0.0000293	0.01375
positive regulation of catalytic activity	10 out of 250 genes, 4.0%	96 out of 10793 genes, 0.9%	0.0000729	0.0342
nucleosome assembly	7 out of 250 genes, 2.8%	46 out of 10793 genes, 0.4%	0.0000813	0.03817
chromatin assembly	7 out of 250 genes, 2.8%	46 out of 10793 genes, 0.4%	0.0000813	0.03817
defense response	11 out of 250 genes, 4.4%	117 out of 10793 genes, 1.1%	0.0000822	0.03858
negative regulation of apoptotic process	9 out of 250 genes, 3.6%	79 out of 10793 genes, 0.7%	0.0000832	0.03902
response to external biotic stimulus	11 out of 250 genes, 4.4%	120 out of 10793 genes, 1.1%	0.0001	0.04855
response to other organism	11 out of 250 genes, 4.4%	120 out of 10793 genes, 1.1%	0.0001	0.04855
