# Supplementary material for: Transcriptome Analysis Reveals Increases in Visceral Lipogenesis and Storage and Activation of the Antigen Processing and Presentation Pathway during the Mouth-Opening Stage in Zebrafish Larvae
Source: Int J Mol Sci. 2017 Jul 31;18(8):1634. doi: 10.3390/ijms18081634 (PMC5578024; doi:10.3390/ijms18081634)
Supplement: Supplementary file 1 [file ijms-18-01634-s001.zip › Table S4.pdf]

Table S4 KEGG pathways with high representation of the DEGs

Pathways	All_ profiles	Profile 0	Profile 1	Profile 3	Profile 4	Profile 6	Profile 7	Pathway ID
Antigen processing and presentation	23	2	0	1	4	5	8	ko04612
Arachidonic acid metabolism	19	1	2	1	2	10	3	ko00590
Mebabolic pathways	246	59	26	37	17	38	51	ko01100
Cell cycle	32	9	2	19	0	1	1	ko04110
Linoleic acid metabolism	10	0	0	1	3	4	2	ko00591
Homologous recombination	13	4	0	8	0	0	0	ko03440
Fat digestion and absorption	19	0	0	1	2	6	8	ko04975
Arginine and proline metabolism	24	4	1	3	3	5	5	ko00330
Glutathione metabolism	18	3	1	6	0	3	3	ko00480
alpha-Linolenic acid metabolism	8	0	0	0	1	2	5	ko00592
Protein digestion and absorption	17	1	0	10	2	1	2	ko04974
Purine metabolism	43	15	10	7	0	4	3	ko00230
Fatty acid metabolism	10	5	0	2	0	1	2	ko00071
PPAR signaling pathway	21	1	0	4	0	4	9	ko03320
Galactose metabolism	12	2	1	0	2	3	4	ko00052
Peroxisome	25	1	3	3	2	8	7	ko04146
Proteasome	17	8	2	0	0	1	4	ko03050
Pentose phosphate pathway	12	2	2	0	1	1	4	ko00030
Tyrosine metabolism	11	1	1	2	0	0	6	ko00350
Circadian rhythm-fly	6	0	0	1	1	0	0	ko04711
Steroid biosynthesis	11	2	0	0	0	2	3	ko00100
Histidine metabolism	9	1	0	2	0	2	4	ko00340
p53 signaling pathway	21	7	1	10	1	2	0	ko04115
Bile secretion	19	4	1	1	2	3	4	ko04976
ECM-receptor interaction	19	5	0	12	0	0	1	ko04512
Drug metabolism-cytochrome P450	12	1	1	1	1	3	4	ko00982
Sttarch and sucrose metabolism	12	3	1	1	3	3	1	ko00500
Terpenoid backbone biosynthesis	8	0	3	4	0	0	0	ko00900
Base excision repair	11	6	2	2	1	0	0	ko03410
Ribosome biogenesis in eukaryotes	20	1	2	0	6	0	1	ko03008
ABC transporters	11	0	1	0	1	3	6	ko02010
Folate biosynthesis	7	2	1	0	0	1	2	ko00790
Circadian rhythm-mammal	7	1	0	1	1	0	0	ko04710
Drug metabolism-other enzymes	11	2	2	1	1	2	2	ko00983
Carbohydrate digestion and absorption	14	1	2	2	3	3	3	ko04973
Fructose and mannose metabolism	10	1	1	1	2	1	4	ko00051
