# Supplementary material for: Transcriptome Analysis Reveals Increases in Visceral Lipogenesis and Storage and Activation of the Antigen Processing and Presentation Pathway during the Mouth-Opening Stage in Zebrafish Larvae
Source: Int J Mol Sci. 2017 Jul 31;18(8):1634. doi: 10.3390/ijms18081634 (PMC5578024; doi:10.3390/ijms18081634)
Supplement: Supplementary file 1 [file ijms-18-01634-s001.zip › Table S5.pdf]

Table S5 Primer sequences of the candidate genes and reference gene for qRT-PCR

Gene name	Accession number	Forward primer (5'→3')	Reverse primer (5'→3')
psme1	ENSDARG00000002165	GAAAATCACCAAGGAGGCTG	TCTTTCTTTCCCTCTTTGGC
hsp90aa1.1	ENSDARG00000010478	AAACAGAGAGAAAAAGAGGTGGA	CTCAAAAAGGTCAAAAGCGG
tap1	ENSDARG00000079766	GTGGGGTTGGCACTCTTG	ACAAAGAAGAAGACGGCTGC
mhc1zba	ENSDARG00000036588	AGTGT GTGGACTGGCTCAAC	CAGGCAGTGTAATGCGGTA
lgmn	ENSDARG00000039150	CAGTGGAGCCAGTGTCGTTG	GAGCGTGGATTTCATTCAGGTA
cd74a	ENSDARG00000009087	GCCCCATTTACTCCAACCAG	GCCTCACCTCACGCTCAGAC
ctssb	ENSDARG00000101051	TGTGGATTCGGCTGTTCTG	CCTGTTTGTAGGGGACGCT
mhc2dab	ENSDARG00000079105	GTGGGGAAGTTTGTGGGGTA	CTGCCTTCAGCCTGCCTC
actb2	ENSDARG00000037870	GCATCACACCTTCTACAACGA	CAGAGTCCATCACGATACCAGT
