# Supplementary figures and images for: Transcriptome Analysis Reveals Increases in Visceral Lipogenesis and Storage and Activation of the Antigen Processing and Presentation Pathway during the Mouth-Opening Stage in Zebrafish Larvae
Source: Int J Mol Sci. 2017 Jul 31;18(8):1634. doi: 10.3390/ijms18081634 (PMC5578024; doi:10.3390/ijms18081634)

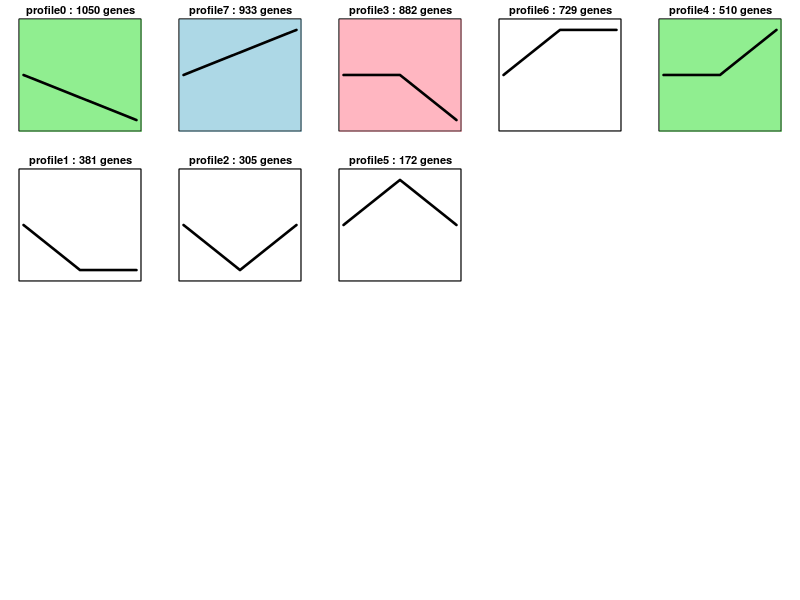

Supplement: Supplementary file 1 [file ijms-18-01634-s001.zip › Fig. S1.tif]
